# Tumour necrosis factor-α induces macromolecule translocation in HIV-derived duodenal organoids

**DOI:** 10.3389/fimmu.2025.1563702

**Published:** 2025-03-18

**Authors:** Kopano Valerie Masete, Alain S. Massarani, Jörg-Dieter Schulzke, Hans-Jörg Epple, Nina A. Hering

**Affiliations:** ^1^ Department of Gastroenterology, Rheumatology and Infectious Diseases, Clinical Physiology/Nutritional Medicine, Charité – Universitätsmedizin Berlin, Berlin, Germany; ^2^ Department of Gastroenterology, Rheumatology and Infectious Diseases, Charité – Universitätsmedizin Berlin, Berlin, Germany; ^3^ Antibiotic Stewardship Team, Medical Directorate, Charité – Universitätsmedizin Berlin, Berlin, Germany; ^4^ Department of General and Visceral Surgery, Charité – Universitätsmedizin Berlin, Berlin, Germany

**Keywords:** HIV, organoids, TNF-α, macromolecule uptake, microbial translocation, transcytosis, paracellular passage, barrier function

## Abstract

**Background:**

Disease progression from human immunodeficiency virus (HIV) infection to acquired immunodeficiency syndrome (AIDS) is marked by chronic immune activation, partly due to increased translocation of gut-derived microbial antigens. Elevated mucosal tumour necrosis factor-α (TNF-α) and resulting epithelial cell apoptosis may be the etiology. However, studies using carcinoma cell lines have failed to find a causal link, possibly due to cellular abnormalities related to the malignant transformation of these immortal cell lines.

**Methods:**

We established intestinal organoid monolayers from healthy controls and HIV-infected adults and characterized their growth dynamics and cellular composition. We then examined the effects of HIV-associated cytokines on transepithelial resistance (TER), apoptosis and macromolecule translocation.

**Results:**

Organoid monolayers from HIV-infected patients grew similarly to healthy controls, forming confluent monolayers within one to two weeks containing enterocytes, Paneth, goblet and stem cells. IFN-γ synergized with TNF-α, allowing TNF-α to cause caspase-mediated apoptosis and TER reduction within 5 ± 3 hours, reflecting patient sample heterogeneity. This led to paracellular passage of 4 kDa Dextran and transcytosis of 44 kDa horse radish peroxidase, both of which could be blocked by pan-caspase inhibitor, Q-VD-Oph.

**Conclusion:**

Our study confirms that intestinal organoid monolayers from biopsies of HIV-infected individuals can be used to study apoptosis-related epithelial barrier dysfunction and macromolecular translocation. We provide direct evidence that TNF-α-induced apoptosis triggered two pathways of macromolecular translocation: paracellular passage via apoptotic leaks and transcytosis. Therapies targeting apoptosis may be useful in preventing disease progression from HIV to AIDS.

## 1 Introduction

Despite the immense success of antiretroviral therapy in preventing human immunodeficiency virus (HIV) transmission and HIV-related deaths, as of 2024, an estimated 9 million people were still living with untreated HIV infection and are at risk of dying from acquired immunodeficiency syndrome (AIDS) (1). Disease progression from HIV infection to AIDS is marked by chronic immune activation, which can also occur in virally suppressed HIV-treated patients (2). Elevated levels of circulating microbial antigens during HIV infection have been shown to drive this immune activation (3–6). Increased uptake of microbial antigens across the gut mucosa, a process termed microbial translocation, has been hypothesized as one of the possible reasons behind these circulating microbial antigens (3, 7, 8). In agreement with this model, we previously found increased macromolecule translocation across the small and large intestinal mucosa of HIV-infected patients (9). However, the possible mechanisms of microbial translocation are still largely unknown. Several cytokines such as tumour necrosis factor-α (TNF-α) and interleukin-4 (IL-4) are elevated in the gut mucosa during HIV infection (10). Gut mucosal apoptosis is high in untreated HIV infection and only partially mitigated by treatment (10–12), strongly implicating TNF-α as it is notoriously known for causing apoptosis (13). This provides a possible mechanism for translocation via apoptotic leaks. However, the role of apoptosis in causing microbial translocation is yet to be confirmed as previous studies, mainly using carcinoma cell lines, have yielded conflicting results (14).

To solve this question, suitable experimental models are needed, that allow for controlled epithelial barrier function perturbation via apoptosis induction and simultaneous measurement of macromolecule translocation. Traditionally, carcinoma cell lines such as T84 (15), Caco2 (16) or HT-29/B6 (17) were used for this purpose; however, they have failed to find a causal link between epithelial apoptosis and macromolecular translocation (14). Carcinoma cell lines lack cellular diversity and various active transport pathways found in the gut (18–24). Most importantly, they have cancer-associated mutations that make them less susceptible to cell death (25) and thus fall short as models for studying cell death. Animal models of lentiviral infection are also not ideal as HIV is so highly specific to humans that even close relatives like nonhuman primates cannot fully recapitulate the disease progression of HIV infection. Finally, using patient-derived intestinal biopsies is also not feasible for these kinds of experiments because their limited survival *in vitro* precludes the long incubation times needed for experimental induction of apoptosis.

To overcome the limitations of the aforementioned experimental models, we opted to establish human organoids as a new model for studying intestinal barrier function in HIV infection. Given the right culture conditions, adult stem cells self-organize into mini 3D organ-like structures *in vitro* known as organoids (26). Organoids can be derived from patients, passaged long-term and/or frozen and thawed as needed, and recapitulate the diverse cellular composition of the tissue from which they originate, making them superior over animal, primary tissue and carcinoma cell line models, respectively. Therefore, the establishment of the human-derived organoid model represents a significant improvement in the experimental armamentarium for studying intestinal barrier function.

As traditional three-dimensional (3D) organoids have a lumen-facing apical side that is experimentally hard to access, we digested 3D organoids to single cells and seeded them in 2D as organoid monolayers on Transwell permeable filter inserts. In this paper, we present for the first time, human intestinal organoids derived from healthy controls and HIV-infected adults. We first confirmed whether HIV-derived organoid monolayers could become confluent and differentiate into various duodenum-specific cells. Based on previous data (9), we hypothesized a link between cytokine-induced apoptosis and macromolecule translocation. We therefore analysed the effect of TNF-α on transepithelial resistance (TER), apoptosis and macromolecule translocation via paracellular passage and transcytosis.

## 2 Materials and methods

### 2.1 Patient samples

This study was performed in compliance with the Ethics Committee of Charité-Universitätsmedizin Berlin (EA4/015/13). Written informed consent was obtained from each patient. Patients were undergoing endoscopy for diagnostic evaluation of gastrointestinal symptoms, unexplained anaemia or for ruling out neoplastic disease. Duodenal biopsies were taken with 3.4 mm biopsy forceps from three HIV-infected patients treated with combination antiretroviral therapy (HIV-treated), one patient who had just been diagnosed with HIV (HIV-untreated), and five healthy controls (**Table 1**). Organoids were immediately generated using up to five biopsies. Organoids could be generated with the same efficiency, whether derived from healthy controls or HIV-infected patient biopsies. Data was acquired for the most optimal growing organoid line from each group and repeated for at least one other line in at least two independent experiments. Some of the healthy control organoid lines were previously characterized in Masete et al. (BMC Biology, 2025) (24); all data presented in this study were generated independently in subsequent experiments.

**Table 1:** Patient clinical data.

Group	Organoid line	Sex	Age	Viral load (copies/mL)	CD4 Count (per µL)	Years on ART	ART
Healthy Control	1C1	M	30	ND	ND	–	–
Healthy Control	3C2	M	53	ND	ND	–	–
Healthy Control	4C3	F	37	ND	ND	–	–
Healthy Control	5C4	F	32	ND	ND	–	–
Healthy Control	6C5	F	35	ND	ND	–	–
HIV-treated	7T1	M	25	<LOD	520	1	3TC, DGV
HIV-treated	8T2	M	84	<LOD	280	27	ABC, 3TC, DGV
HIV-treated	9T3	M	54	<LOD	980	16	TAF, FTC, BGV
HIV-untreated	2U1	M	41	1216000	110	–	–

ART, Antiretroviral therapy; 3TC, lamivudine; ABC, abacavir; BGV, bictegravir; DGV, dolutegravir; FTC, emtricitabine; F, female; M, male; ND, not determined, TAF, tenofovir alefenamide; <LOD, lower than the limit of detection (<20 copies/mL).

### 2.2 Organoid culture

Organoids were cultured as previously described (24). Briefly, biopsies were processed to isolate crypts, which were then embedded in 50 μL Cultrex™ BME (R&D Systems) in 24-well plates (TPP/Merck). 3D organoids were cultured in 3D medium (defined in the **Supplementary File**) and passaged an average of 1:4 wells weekly. To generate 2D organoids (organoid monolayers), 3D organoids were digested into single cells using TrypLE™ Express (Gibco/Thermo Fisher Scientific) for 10 minutes at 37°C. Around 5·10^5^ cells were seeded on uncoated Transwell filter inserts (Millipore) and immediately differentiated using 2D medium (defined in the **Supplementary File**). TER at 37°C was monitored using a chopstick electrode (STX2, World Precision Instruments) and corrected by subtracting the resistance of cell-free Transwells (130 Ω) and multiplied by the effective area of the Transwells (0.6 cm^2^).

### 2.3 Apoptosis and inhibitor assays

Confluent monolayers were basolaterally stimulated with human TNF-α (Peprotech/Thermo Fisher Scientific), always after 72h basolateral pre-stimulation with 1 ng/mL human interferon-gamma (IFN-γ, Peprotech). In initial experiments, stimulation with IL-4 (Peprotech) alone or in combination with IFN-γ and TNF-α was also carried out. Unstimulated negative controls were included in each experiment, henceforth simply referred to as “Control”. Caspase 3/7 activity was measured using the SensoLyte Homogenous AFC Caspase-3/7 Assay Kit (Anaspec) according to manufacturer’s instructions. Apoptosis was blocked with Quinoline-Val-Asp-Difluorophenoxymethylketone (Q-VD-Oph, MedChemExpress/Hycultec), which was added apically and basolaterally, 18h before (overnight) and during TNF-α stimulation. Transcytosis was blocked with 40 µM Dynasore (Enzo Biochem), which was added apically and basolaterally, 30 minutes before and during permeability measurements. Negative controls were treated with dimethyl sulfoxide (DMSO) in the same manner during the Q-VD-Oph or Dynasore experiments.

### 2.4 Protein and mRNA expression

For the real-time quantitative PCR (RT-qPCR), total RNA was extracted with the NucleoSpin™ RNA/Protein Purification Mini Kit (Macherey-Nagel) and reverse transcribed with the High-Capacity RNA-to-cDNA Kit (Applied Biosystems/Thermo Fisher Scientific) according to the manufacturer’s protocol. RT-qPCR reactions were performed using a QuantStudio 3 thermocycler (Applied Biosystems) with 2 μg of cDNA template, 1 μL of each probe, 10 μL of RT-qPCR Master Mix (Applied Biosystems) and was made up to 20 μl using nuclease-free water. GAPDH (Applied Biosystems) was used for normalization following the 2^-ΔCT^ method (27). Immunofluorescent staining (28) and Western blots (29) were performed according to previous publications. Human probes and antibodies used can be found in the **Supplementary File**.

### 2.5 Permeability measurements

Unless otherwise stated, permeability measurements were conducted after TER decreased to around 130-200 Ω·cm^2^ following 5 ± 3h 5 ng/mL (100 units/mL) TNF-α stimulation, based on each organoid line’s sensitivity to TNF-α. Permeability measurements were performed at 37°C in 24 well plates (TPP/Merck) in 10 mM glucose-enriched, pH 7.4, HEPES-buffered Ringer solution (plate assay). In all plate assays, 0.4 mM dialyzed fluorescein isothiocyanate-labelled 4 kDa dextran (FD4, TdBConsultancy) and 25 nM 44 kDa Horse Radish Peroxidase (HRP, Sigma-Aldrich/Merck) was added to each Transwell (apical solution). To eliminate the concentration gradient, 0.4 mM unlabelled dextran was added to each well (basolateral solution). FD4 and HRP permeability was measured by transferring Transwells from one well to another at 0 (blank), 30, 60 and 90 minutes. HRP was quantified using a fluorogenic peroxidase substrate kit (Quanta Blu™, Thermo Fisher Scientific) and, along with FD4, detected fluorometrically using a plate reader (Infinite M200, Tecan). Permeabilities were calculated as the ratio of flux, J (mol·h^-1^·cm^-2^) over concentration gradient, Δc (mol/L). Initial experiments were performed in Ussing chambers as described before (9), yielding qualitatively identical macromolecule permeabilities as plate assays.

### 2.6 Statistics

GraphPad Prism (version 10) was used for graphing and statistical analysis. Results are given as mean ± SEM. Multivariate analysis was performed using one-way or two-way ANOVA. Bonferroni–Holm adjustment was used for *post hoc* analysis in multiple testing. Adjusted P-values < 0.05 were considered significant.

## 3 Results

### 3.1 HIV-derived organoid monolayers are comparable to those of healthy controls

**Figure 1:**
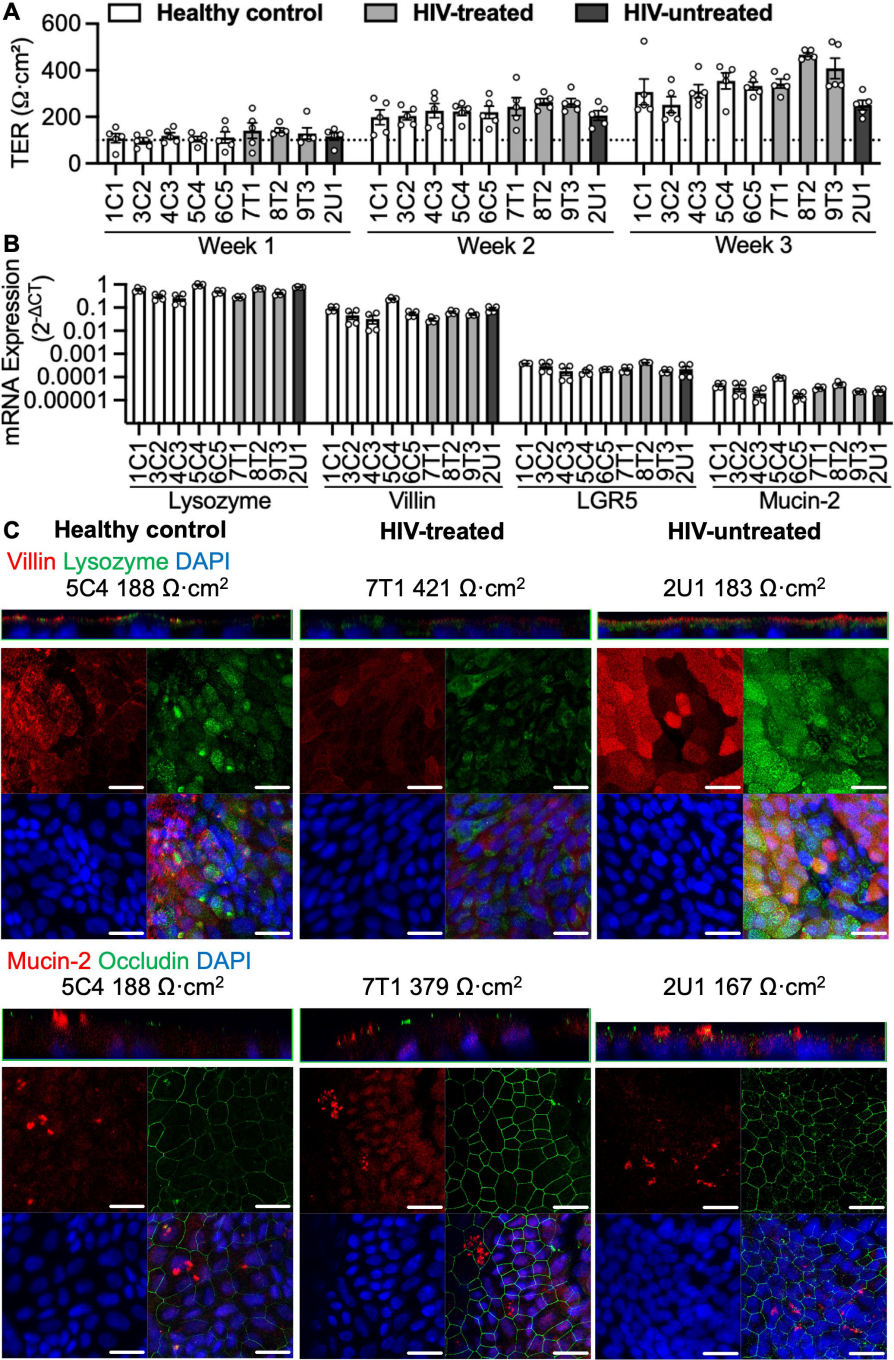
Characterising HIV-derived organoid monolayers. **(A)** Growth kinetics of organoid monolayers, n = 5. **(B)** Relative mRNA expression (normalized to GAPDH, log10-transformed) of major duodenum cell markers, n = 4. **(C)** Z-axis and confocal projections of organoid monolayers stained for enterocytes (Villin) and Paneth cells (Lysozyme), Goblet cells (Mucin-2) and tight junction marker (occludin). Scale bar, 20 μm.

3D organoids derived from five healthy controls, three HIV-treated and one HIV-untreated patient were digested to single cells and seeded as organoid monolayers. Irrespective of their source, organoid monolayers reached confluence within one to two weeks (TER ≥ 100 Ω·cm^2^, marked by a dotted line, **Figure 1A**). This TER threshold for confluence is consistent with a previous report (30) and corresponds to the lowest TER value where apical medium volume consistently remained unchanged for at least two days. TER increased on average by 108.7 ± 0.1 Ω·cm^2^ weekly, with an average increase of 217.4 ± 19.9 Ω·cm^2^ from week 1 to 3 and no TER differences between healthy control and HIV-infected patient organoids (**Figure 1A**, **Supplementary Table S1**). Organoid monolayers of all 9 lines had an abundance of lysozyme (Paneth cell marker) and Villin (enterocyte marker) and modest expression of Mucin-2 (Goblet cell marker) and LGR5 (stem cell marker) mRNA transcripts (**Figure 1B**). Immunofluorescence staining showed protein expression of Villin, Lysozyme and Mucin-2 localized towards the apical side of healthy control, HIV-treated and HIV-untreated organoid monolayers (**Figure 1C**). This was confirmed in one other healthy control and HIV-treated line (**Supplementary Figure S1**). There were no qualitative differences in epithelial integrity based on occludin staining of healthy control, HIV-treated and HIV-untreated organoid monolayers (**Figure 1C**, **Supplementary Figure S1**). There were numerous significant differences between mRNA transcript levels of different organoid lines (**Supplementary Table S2**). However, qualitative protein expression of cell markers did not differ between organoid monolayers obtained from healthy controls, HIV-treated and HIV-untreated patients (**Figure 1C**, **Supplementary Figure S1**). Further experiments were conducted using three-week-old organoid monolayers, as organoid monolayers had the highest TER at this time point, using at least two HIV-derived monolayers and the two most optimal-growing healthy control lines (5C4 and 6C5, **Supplementary Table S1**).

### 3.2 TNF-α causes epithelial barrier defects in IFN-γ-stimulated organoid monolayers

Next, we investigated the effects of HIV-associated cytokines, TNF-α, IFN-γ and IL-4, on organoid monolayers. It has long been reported that IFN-γ increases TNF-α receptor mRNA levels (31), cell-surface expression (32) and binding (33) in cancer cell lines. We found that a 72h pre-stimulation with 1 ng/mL IFN-γ was necessary to see a TNF-α-induced TER reduction (**Supplementary Figure S2A**). We confirmed in Ussing chamber experiments that this small dose of IFN-γ neither reduced TER nor induced macromolecular permeability (**Supplementary Figure S2B**). IFN-γ merely sensitized organoid monolayers to TNF-α, not by changing TNF-α receptor protein expression levels (**Supplementary Figure S2C**), but rather by relocating the TNF-α receptor from the intracellular space to the apical cell surface as shown by immunofluorescence staining (**Supplementary Figure S2D**).

**Figure 2:**
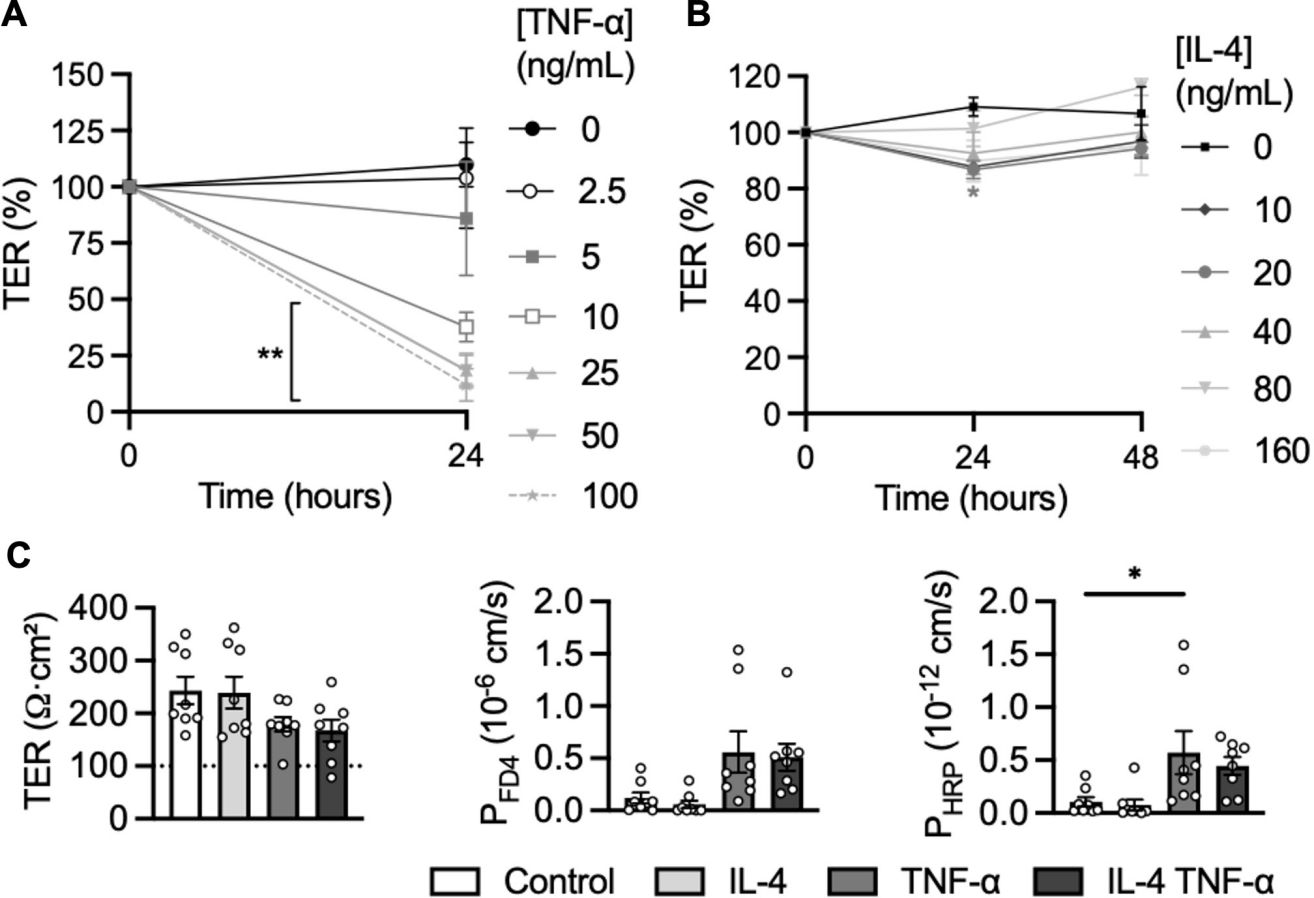
Effects of HIV-associated cytokines on epithelial barrier function. Effects of different concentrations of **(A)** TNF-α and **(B)** IL-4 on TER, n = 4. **(C)** Effects of 20 ng/mL IL-4 and/or 5 ng/mL TNF-α stimulation for 24h on TER, FD4 and HRP translocation, n = 8. Data was obtained from various patient lines. *p<0.05 and **p<0.01.

To preclude unspecific effects of unphysiologically high cytokine concentrations, we wanted to identify the lowest effective concentration of TNF-α. In dose-response experiments, we found the effect of TNF-α on TER started to unfold at a concentration of 5 ng/ml (**Figure 2A**). Unlike TNF-α, IL-4 (20 ng/mL) only modestly and transiently altered TER after 24h (p = 0.0375, **Figure 2B**). Therefore, organoid monolayers were stimulated with 5 ng/mL TNF-α (following IFN-γ pre-stimulation) and/or 20 ng/mL IL-4 for 24h. Only TNF-α but not IL-4 caused modest TER reduction sufficient to induce FD4 (p = 0.0515) and HRP (p = 0.0231) permeability in plate assays (**Figure 2C**). All further experiments were conducted without IL-4, with 5 ng/mL TNF-α following IFN-γ pre-stimulation, in plate assays.

### 3.3 Low-level TNF-α-induced apoptosis triggers macromolecular translocation

**Figure 3:**
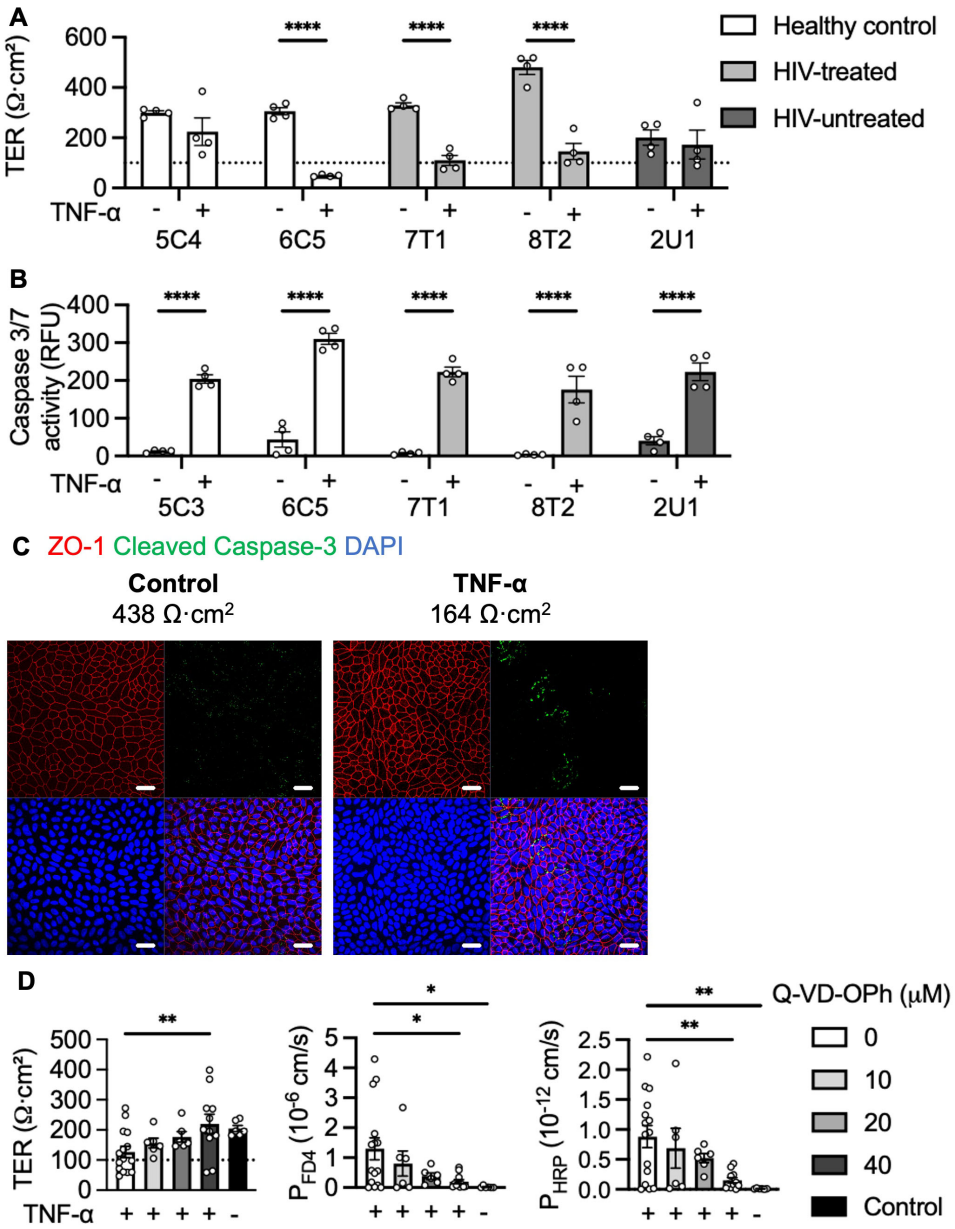
TNF-α-induced low-level apoptosis triggers macromolecule translocation. Effects 5 ng/mL 5h TNF-α stimulation on **(A)** TER and **(B)** caspase activity, n = 4. **(C)** Confocal projections of tight junction marker (ZO-1) and cleaved caspase-3 of representative organoid monolayers following 5 ng/mL 5h TNF-α stimulation. Scale bar, 20 μm. **(D)** Apoptosis was blocked with Q-VD-Oph overnight before 5 ng/mL TNF-α stimulation and TER, FD4 and HRP translocation was measured. An unstimulated control was included. n = 6-15, obtained from various patient lines. *p<0.05, **p<0.01 and ****p<0.0001.

To measure early apoptotic events, we investigated whether low-level apoptosis could be induced by a 5h TNF-α stimulation. TNF-α reduced TER of most organoid lines, with 6C5 (healthy control) TER being drastically reduced below the threshold of confluence (**Figure 3A**). Simultaneously, TNF-α increased caspase activity by at least 5.5-fold (p < 0.0001), with 6C5 (healthy control) and 8T2 (HIV-treated) having the highest and lowest caspase 3/7 activity, respectively (**Figure 3B**). Epithelial integrity was assessed by immunofluorescence staining. Compared to the unstimulated control, 5 ng/mL stimulation did not affect the overall epithelial integrity of the organoid monolayers (**Figure 3C**). To confirm that it was in fact apoptosis that caused the TNF-α-induced macromolecular permeability seen in **Figure 2C**, Q-VD-OPh, a pan-caspase inhibitor, was used to block apoptosis induced by 5 ng/mL TNF-α. Pre-treatment with 40 µM Q-VD-Oph prevented the TNF-α-induced reduction in TER (p = 0.0099), FD4 permeability (p = 0.0228) and interestingly, also HRP permeability (p = 0.0050, **Figure 3D**).

### 3.4 Q-VD-Oph blocks TNF-α-induced macromolecular translocation

**Figure 4:**
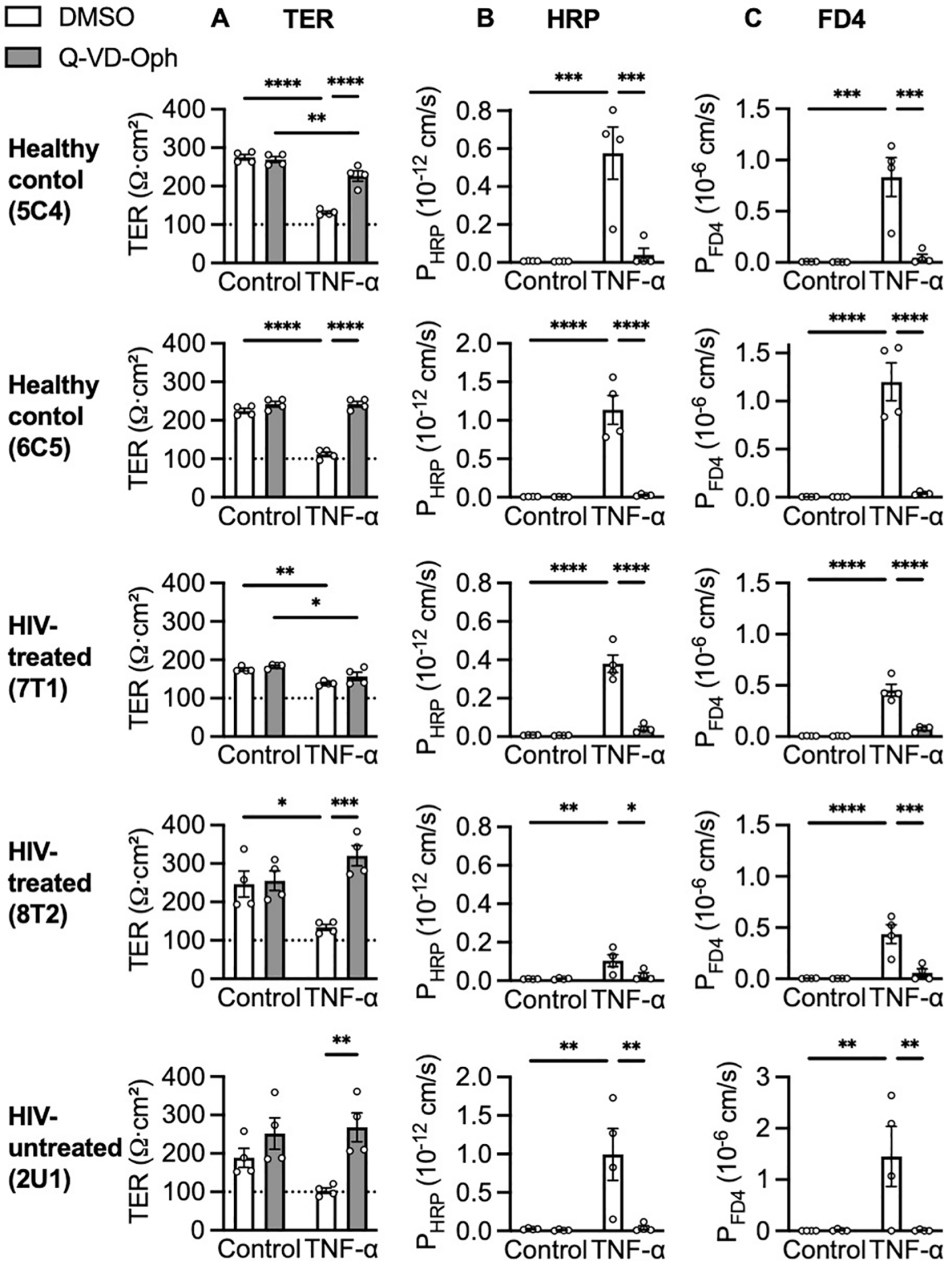
TNF-α-induced macromolecule translocation. Effects of 40 µM overnight Q-VD-Oph pre-treatment on **(A)** TER, **(B)** HRP and **(C)** FD4 translocation measured after 5 ng/mL TNF-α stimulation, n = 4. *p<0.05, **p<0.01, ***p<0.001 and ****p<0.0001

The effects of 40 µM Q-VD-Oph on TNF-α-induced epithelial barrier defects of healthy control, HIV-treated and HIV-untreated organoid monolayers were analysed. Q-VD-Oph generally protected healthy control, HIV-treated and HIV-untreated organoid monolayers against the TNF-α-induced reduction in TER (**Figure 4A**). Furthermore, Q-VD-Oph prevented their respective TNF-α-induced HRP (**Figure 4B**) and FD4 (**Figure 4C**) translocation.

### 3.5 TNF-α induces macromolecular paracellular passage and transcytosis

**Figure 5:**
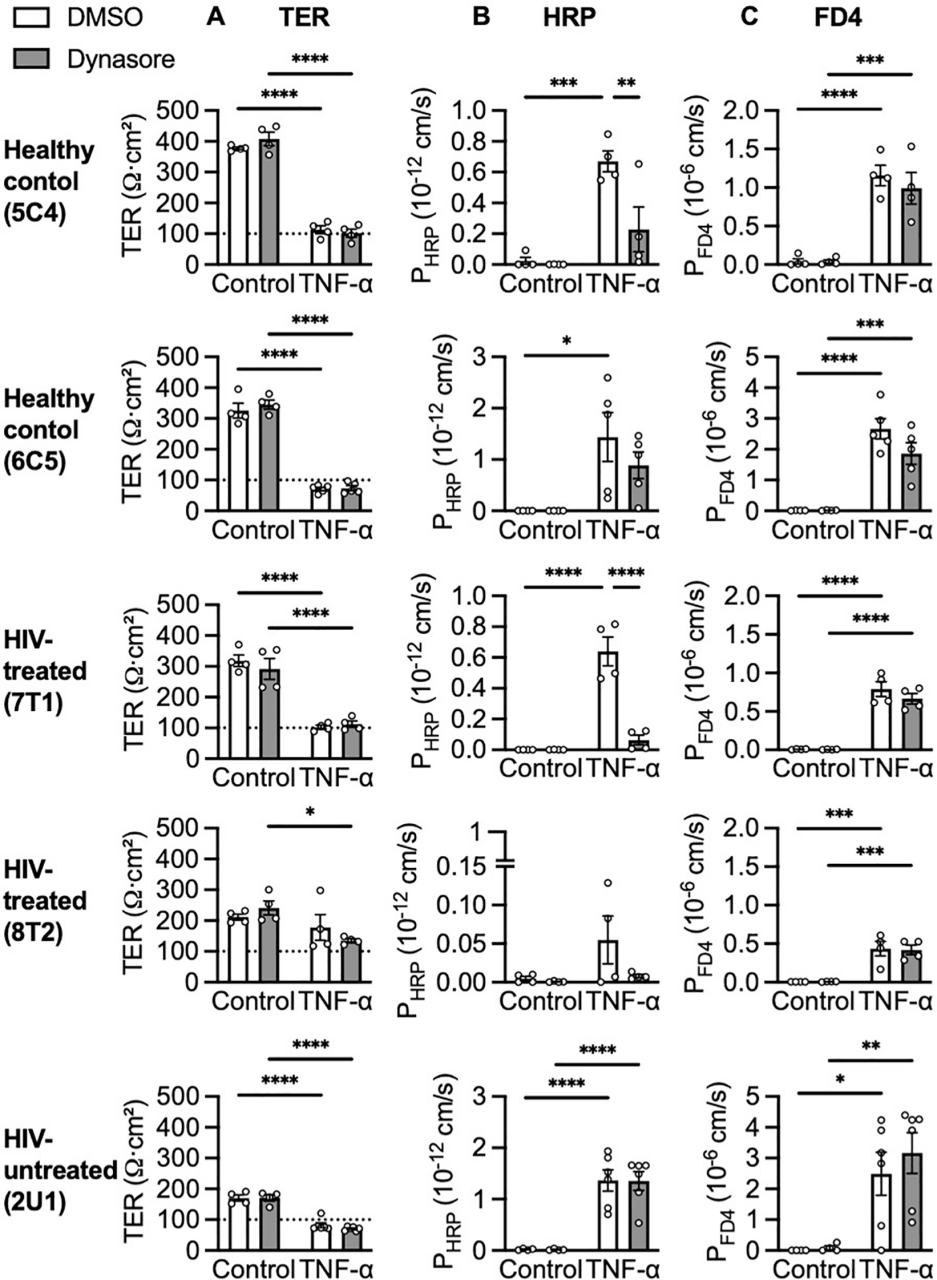
TNF-α-induced transcytosis of large molecules. Effects of 30 minutes pre-treatment with 40 µM Dynasore on **(A)** TER, **(B)** HRP and **(C)** FD4 translocation after 5 ng/mL TNF-α stimulation. n = 4-6. *p<0.05, **p<0.01, ***p<0.001 and ****p<0.0001.

To further elucidate the mechanism of macromolecule translocation caused by TNF-α, organoid monolayers were treated with Dynasore, a molecule known to inhibit endocytosis by inhibiting the activity of dynamin and dispersing the organization of lipids in lipid rafts (34). Without affecting TER (**Figure 5A**), Dynasore blocked HRP (**Figure 5B**) but not FD4 (**Figure 5C**) translocation of 5C4 (healthy control, p = 0.0046) and 7T1 (HIV-treated, p < 0.0001). This implicates transcytosis as well as paracellular passage through apoptotic leaks as mechanisms of macromolecule translocation. For 2U1 (HIV-untreated) and 6C5 (healthy control), a strong TNF-α response led to TER reducing below the confluence threshold (**Figure 5A**), resulting in high macromolecular translocation that could not be blocked by Dynasore (**Figures 5B, C**). Inversely for 8T2 (HIV-treated), a weak TNF-α TER response (**Figure 5A**) resulted in low-level HRP translocation that was modestly but insignificantly reduced by Dynasore (**Figure 5B**, p = 0.0852). Hence, Dynasore could block HRP translocation only when monolayers were intact but sufficiently impaired by TNF-α.

## 4 Discussion

We established intestinal organoids to study epithelial barrier function and macromolecule translocation in HIV infection. Compared to carcinoma cell lines traditionally used for this purpose, our organoid model is a superior representation of the intestinal mucosal barrier as stem cells used for organoid generation were endoscopically obtained from HIV-infected patients and healthy controls. Furthermore, the organoids formed confluent monolayers reflecting all major duodenum-specific cell types and, as shown previously, better reproduce active transport pathways of the intestinal mucosa than carcinoma cell lines (24). Most importantly, it is unknown to what extent the results obtained using carcinoma cell lines were biased by cellular abnormalities related to their malignant transformation as previous studies using carcinoma cell lines failed to find a causal link between epithelial apoptosis and macromolecule translocation (14). In contrast, TNF-α caused caspase-mediated apoptosis that induced paracellular passage and transcytosis of macromolecules in our organoid monolayers. Both pathways of macromolecular translocation could be pharmacologically blocked by pan-caspase inhibitor, Q-VD-Oph, suggesting a link between apoptosis and transcytosis.

In addition to TNF-α, IL-4 is also elevated in the gut mucosa of HIV-infected patients (10). IL-4 increases intestinal macromolecule translocation in T84 cells and mouse models (35). However, the TER and macromolecule translocation of our duodenum-derived organoid monolayers were largely unaffected by basolateral and/or apical stimulation with up to 160 ng/mL IL-4. This was also the case for IL-13, which shares the same receptor as IL-4; furthermore, colon-derived organoid monolayers were similarly unaffected by IL-4 (data not shown). Our organoid monolayers’ insensitivity to IL-4 could be due to low receptor expression, improper localization, or other regulatory factors. This, as well as the role IL-4 may play in intestinal epithelial barrier function, remains an open question. Unlike TNF-α and IL-4, IFN-γ is not elevated in the intestinal mucosa during HIV infection (10) but is involved in the immune response to HIV [reviewed in (36)]. IFN-γ impairs barrier integrity of intestinal organoids (37, 38) and T84 cells (15, 39) at concentrations higher than 10 ng/mL. This was also the case in our hands (data not shown), but we found optimal experimental conditions where a 72h 1 ng/mL (20 units/mL) IFN-γ pre-stimulation merely sensitized organoid monolayers to TNF-α without impairing barrier integrity.

The synergy between IFN-γ and TNF-α in potentiating intestinal barrier disruptions was recently summarized (14). Even though TNF-α is known to cause epithelial apoptosis, the mechanism by which it increases intestinal epithelial permeability has long been solely attributed to apical junction disruptions (15). This was based on many reports that pharmacological inhibition of TNF-α-induced epithelial apoptosis failed to block TNF-α-induced macromolecule translocation [reviewed in (14)]. Most studies with this conclusion were done using carcinoma cell lines like T84 and Caco2 cells, which by definition are immortalized by mutations that select for survival. Mouse (MC38) and human carcinoma (Caco2) cell lines were more resistant to cell death when exposed to the same doses of TNF-α, cytotoxic drugs, and X-ray irradiation compared to mouse-derived organoids (25). Alternatively, the failure of pan-caspase blockers against TNF-α-induced barrier defects in previous studies could be due to the use of less potent, more toxic pan-caspase blockers such as zVAD (40) and/or toxic concentrations of IFN-γ/TNF-α. We used the lowest effective concentration of TNF-α, leading to threshold-dependent responses where some monolayers exhibited a clear TER effect, while others barely had a TER effect. This resulted in considerable variability in TER after TNF-α stimulation across organoid lines and replicates, likely due to differences in initial TER, organoid monolayer cellular composition and TNF-R1 cell-surface expression. However, this low TNF-α dose ensured minimal epithelial disruptions, which were attenuated by Q-VD-Oph in a dose-dependent manner. The highest Q-VD-Oph concentration (40 µM) significantly blocked TNF-α-induced TER reduction and macromolecule translocation, even for the most TNF-α-sensitive organoid lines.

2U1 (HIV-untreated) and 6C5 (healthy control) were the most responsive to TNF-α with as little as 2h TNF-α stimulation resulting in a rapid TER reduction and robust macromolecular translocation that could not be blocked by Dynasore, thus likely occurring primarily via apoptotic leaks. On the other hand, translocation of HRP occurred mainly via transcytosis in intact monolayers of 7T1 (HIV-untreated) and 5C4 (healthy control) following 5h TNF-α stimulation. Abrogation of transcytosis by Q-VD-Oph suggests that apoptosis triggered transcytosis. This follows our previous observations of transcytosis induction in T84 cells by apoptosis inducer, camptothecin (9). During early apoptosis, elevated adenosine triphosphate levels cause accelerated intracellular transport (41), providing a possible reason for elevated transcytosis following apoptosis initiation.

High TNF-α receptor expression in Western blots of 6C5 and 2U1 offers an explanation for their high TNF-α sensitivity. 8T2 (HIV-treated) was the least responsive to TNF-α, leading to barely any TER reduction, caspase activity nor macromolecular translocation even after 8h stimulation. 8T2 organoids were derived from a patient classified as an ART-suppressed immunologic nonresponder [<350 CD4 cells/µL (42)] and interestingly, time on treatment seemed to negatively correlate with TNF-α sensitivity (8T2, 27 years; 7T1, 1 year, and 2U1, untreated). TNF-α also causes intestinal epithelial apoptosis in Crohn’s disease (43) and interestingly, colon-derived organoid monolayers from Crohn’s disease patients have impaired epithelial integrity compared to healthy controls, with those from patients treated with anti-TNF-α antibody (adalimumab) tending to be less sensitive to bacteria-induced barrier defects (44). While this study (44) and ours were limited by small sample size, we speculate that these findings may reflect the *in vivo* state as a separate study reported that Crohn’s disease-associated epigenetic alterations (DNA methylation patterns) present in intestinal biopsies could be retained in organoids (45). Given that T helper cell cytokine signalling has been shown to modulate intestinal stem cell fate (46), it appears plausible that chronic inflammation during HIV infection may similarly induce epigenetic alterations that might alter epithelial function.

In summary, we present for the first time intestinal organoids derived from HIV-infected patients. While growth dynamics and cellular composition did not differ, significant differences in TNF-α sensitivity were observed in different organoid lines warranting future studies with larger sample sizes to investigate differences between HIV patient groups. TNF-α reduced TER, caused apoptosis and triggered macromolecule translocation paracellular passage via apoptotic leaks and transcytosis were identified as translocation pathways. It would be valuable to investigate the translocation of biologically relevant macromolecules, such as lipopolysaccharides in future studies. Therapies targeting epithelial apoptosis may be useful in preventing disease progression from HIV to AIDS (47). Perhaps even for other pathologies such as Crohn’s disease where TNF-α also causes apoptosis.

## Data Availability

The raw data supporting the conclusions of this article will be made available by the authors, without undue reservation.
