# Supplementary material for: Tumour necrosis factor-α induces macromolecule translocation in HIV-derived duodenal organoids
Source: Front Immunol. 2025 Mar 18;16:1563702. doi: 10.3389/fimmu.2025.1563702 (PMC11959035; doi:10.3389/fimmu.2025.1563702)
Supplement: Supplementary file 1 [file DataSheet1.pdf]

Supplementary Material

1 Supplementary Data

Human probes and antibodies

The probes used for quantitative real-time PCR (RT-qPCR) were all from Thermo Fisher Scientific: Lysozyme (LYZ, Hs00426232_m1), Villin (VIL1, Hs01031739_m1), Mucin-2 (MUC2, Hs00894025_m1) and Leucine-rich repeat-containing G-protein coupled receptor 5 (LGR5, Hs00969422_m1).

The antibodies used for immunofluorescent staining were rabbit anti-lysozyme (1:100, F0372, Dako), goat anti-Villin (1:100, SC-7672, Santa Cruz), rabbit anti-Mucin-2 (1:50, SC-15334, Santa Cruz), rabbit anti-Occludin (1:100, 71-1500, Invitrogen), mouse anti-Zonula Occludens-1 (ZO-1, 1:100, 610966, BD Biosciences), rabbit anti-cleaved Caspase-3 (1:200, 9964, Cell Signalling), rabbit anti-TNFRSF1A (1:50, HPA004102, Atlas Antibodies), and DAPI (1:1 000, 10 236 276 001, Roche). Images were obtained using a confocal laser scanning microscope (LSM 780, Carl Zeiss AG).

The antibodies used for Western blots were goat anti-TNF-R1 (1:1 000, SC-1067, Santa Cruz) and mouse anti-β-Actin (1:20 000, A5441, Sigma Aldrich).

3D and 2D Medium

The base medium component was Advanced Dulbecco's modified Eagle medium (DMEM)/F12 (Gibco) with 10 mmol/L HEPES (Gibco), 1x Glutamax (Gibco), 1% 100 U/ml penicillin and 1% 100 mg/ml streptomycin. 3D medium was produced from stable transfected HEK293 cells (ATTC) conditioned medium with 50 % L-WRN [1], 20 % Rspo1 [2] and 10 % Noggin [3] supplemented with 50 ng/ml human epithelial growth factor (Peprotech), 1 mM N-acetylcysteine (Sigma-Aldrich), 10 mM nicotinamide (Sigma-Aldrich), 1 μM SB-202190 (Stemcell), 500 nM A83-01 (Stemgent), 1x B27 (Gibco) and 1x N2 (Gibco). 2D medium was identical to 3D medium in composition and concentrations but was without L-WRN, SB-202190 and A83-01.

2 Supplementary Figures and Tables

Table S1. Two-way ANOVA, Bonferroni's multiple comparisons test of Figure 1A

Comparison	Difference ($\Omega \cdot cm^2$)	Summary	Adjusted P Value
Week 3			
2U1 vs. 8T2	-216.6	**	0.0063
6C5 vs. 8T2	-135.3	*	0.0186
7T1 vs. 8T2	-123.9	*	0.0484
1C1			
Week 1 vs. 2	-90.09	*	0.0422
2U1			
Week 1 vs. 2	-89.64	*	0.0102
Week 1 vs. 3	-134	**	0.0085
Week 2 vs. 3	-44.37	*	0.0407
3C2			
Week 1 vs. 2	-107.2	*	0.0118
Week 1 vs. 3	-154.7	*	0.0104
4C3			
Week 1 vs. 2	-107.8	*	0.0111
Week 1 vs. 3	-191.4	**	0.0012
Week 2 vs. 3	-83.67	*	0.0254
5C4			
Week 1 vs. 2	-119.3	**	0.0095
Week 1 vs. 3	-250.7	**	0.0079
Week 2 vs. 3	-131.4	*	0.0112
6C5			
Week 1 vs. 2	-108.1	**	0.0043
Week 1 vs. 3	-221	***	0.0001
Week 2 vs. 3	-113	*	0.0133
7T2			
Week 1 vs. 2	-103.1	**	0.0032
Week 1 vs. 3	-202.4	*	0.0124
8T2			
Week 1 vs. 2	-120.9	**	0.0017
Week 1 vs. 3	-324.4	****	<0.0001
Week 2 vs. 3	-203.5	***	0.0007
9T3			
Week 1 vs. 2	-131.6	**	0.001
Week 1 vs. 3	-279.5	**	0.0036

Table S2. Two-way ANOVA, Bonferroni's multiple comparisons test of Figure 1B

Comparison	Difference ($2^{-\Delta CT}$)	Sum-mary	Adjusted P Value	Comparison	Difference ($2^{-\Delta CT}$)	Sum-mary	Adjusted P Value
Lysozyme				1C1			
1C1 vs. 2U1	-15.72	****	<0.0001	Lysozyme vs. Villin	48.59	****	<0.0001
1C1 vs. 3C2	28.08	****	<0.0001	Lysozyme vs. LGR5	57.15	****	<0.0001
1C1 vs. 4C3	32.76	****	<0.0001	Lysozyme vs. Mucin-2	57.18	****	<0.0001
1C1 vs. 5C4	-33.95	****	<0.0001	Villin vs. LGR5	8.55	*	0.0489
1C1 vs. 6C5	11.07	*	0.0252	Villin vs. Mucin-2	8.58	*	0.0474
1C1 vs. 7T1	30.28	****	<0.0001	2U1			
1C1 vs. 9T3	17.76	****	<0.0001	Lysozyme vs. Villin	64.59	****	<0.0001
2U1 vs. 3C2	43.74	****	<0.0001	Lysozyme vs. LGR5	72.88	****	<0.0001
2U1 vs. 4C3	48.48	****	<0.0001	Lysozyme vs. Mucin-2	72.90	****	<0.0001
2U1 vs. 5C4	-18.24	****	<0.0001	3C2			
2U1 vs. 6C5	26.79	****	<0.0001	Lysozyme vs. Villin	24.72	****	<0.0001
2U1 vs. 7T1	46.00	****	<0.0001	Lysozyme vs. LGR5	29.14	****	<0.0001
2U1 vs. 9T3	33.48	****	<0.0001	Lysozyme vs. Mucin-2	29.16	****	<0.0001
3C2 vs. 5C4	-61.97	****	<0.0001	4C3			
3C2 vs. 6C5	-16.95	****	<0.0001	Lysozyme vs. Villin	21.30	****	<0.0001
3C2 vs. 8T2	-33.85	****	<0.0001	Lysozyme vs. LGR5	24.41	****	<0.0001
4C3 vs. 5C4	-66.71	****	<0.0001	Lysozyme vs. Mucin-2	24.42	****	<0.0001
4C3 vs. 6C5	-21.69	****	<0.0001	5C4			
4C3 vs. 8T2	-38.59	****	<0.0001	Lysozyme vs. Villin	68.42	****	<0.0001
4C3 vs. 9T3	-15.00	***	0.0002	Lysozyme vs. LGR5	91.12	****	<0.0001
5C4 vs. 6C5	45.03	****	<0.0001	Lysozyme vs. Mucin-2	91.13	****	<0.0001
5C4 vs. 7T1	64.23	****	<0.0001	Villin vs. LGR5	22.70	****	<0.0001
5C4 vs. 8T2	28.12	****	<0.0001	Villin vs. Mucin-2	22.71	****	<0.0001
5C4 vs. 9T3	51.72	****	<0.0001	6C5			
6C5 vs. 7T1	19.21	****	<0.0001	Lysozyme vs. Villin	40.86	****	<0.0001
6C5 vs. 8T2	-16.91	****	<0.0001	Lysozyme vs. LGR5	46.09	****	<0.0001
7T1 vs. 8T2	-36.11	****	<0.0001	Lysozyme vs. Mucin-2	46.11	****	<0.0001
7T1 vs. 9T3	-12.52	**	0.0051	7T1			
8T2 vs. 9T3	23.59	****	<0.0001	Lysozyme vs. Villin	23.97	****	<0.0001
Villin				Lysozyme vs. LGR5	26.88	****	<0.0001
1C1 vs. 5C4	-14.13	***	0.0007	Lysozyme vs. Mucin-2	26.90	****	<0.0001
2U1 vs. 5C4	-14.41	***	0.0005	8T2			
3C2 vs. 5C4	-18.28	****	<0.0001	Lysozyme vs. Villin	56.87	****	<0.0001
4C3 vs. 5C4	-19.59	****	<0.0001	Lysozyme vs. LGR5	62.98	****	<0.0001
5C4 vs. 6C5	17.47	****	<0.0001	Lysozyme vs. Mucin-2	63.01	****	<0.0001
5C4 vs. 7T1	19.78	****	<0.0001	9T3			
5C4 vs. 8T2	16.57	****	<0.0001	Lysozyme vs. Villin	34.39	****	<0.0001
5C4 vs. 9T3	17.69	****	<0.0001	Lysozyme vs. LGR5	39.41	****	<0.0001
LGR5	No differences			Lysozyme vs. Mucin-2	39.42	****	<0.0001
Mucin-2	No differences						

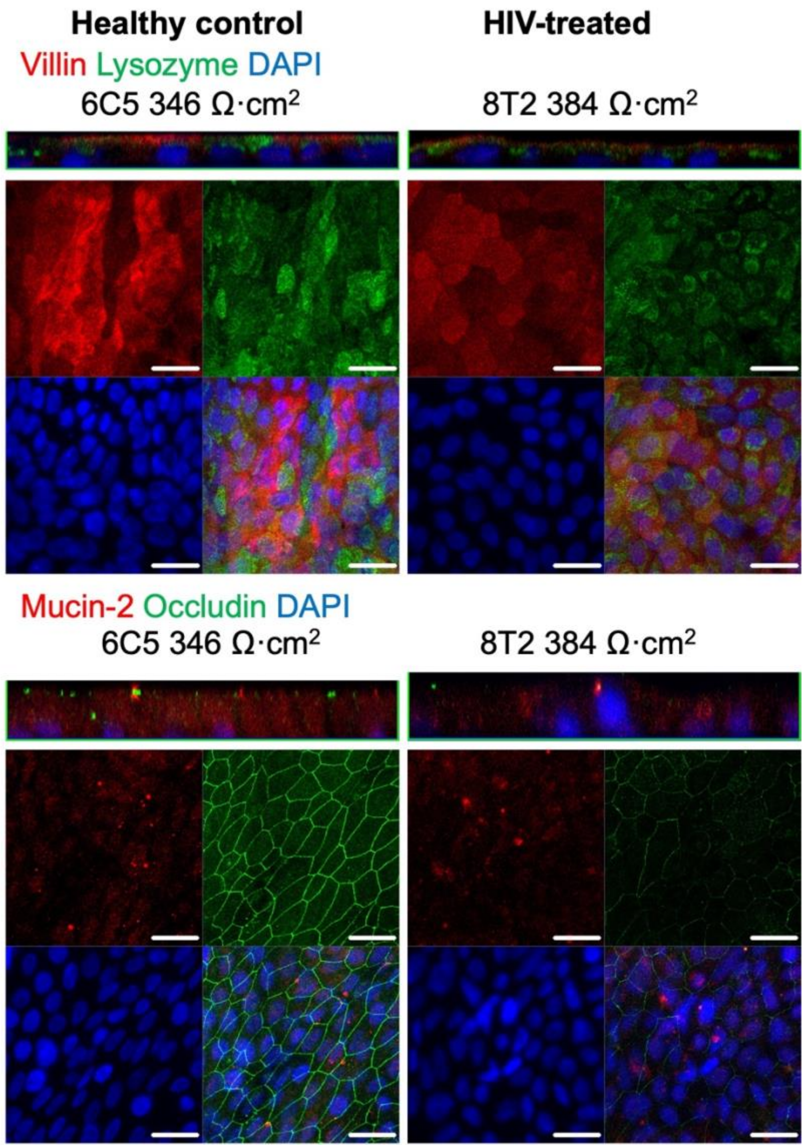

Figure S1. Cell markers. Z-axis and confocal projections of organoid monolayers stained for enterocytes (Villin) and Paneth cells (Lysozyme), Goblet cells (Mucin-2) and tight junction marker (occludin). Scale bar, 20 µm

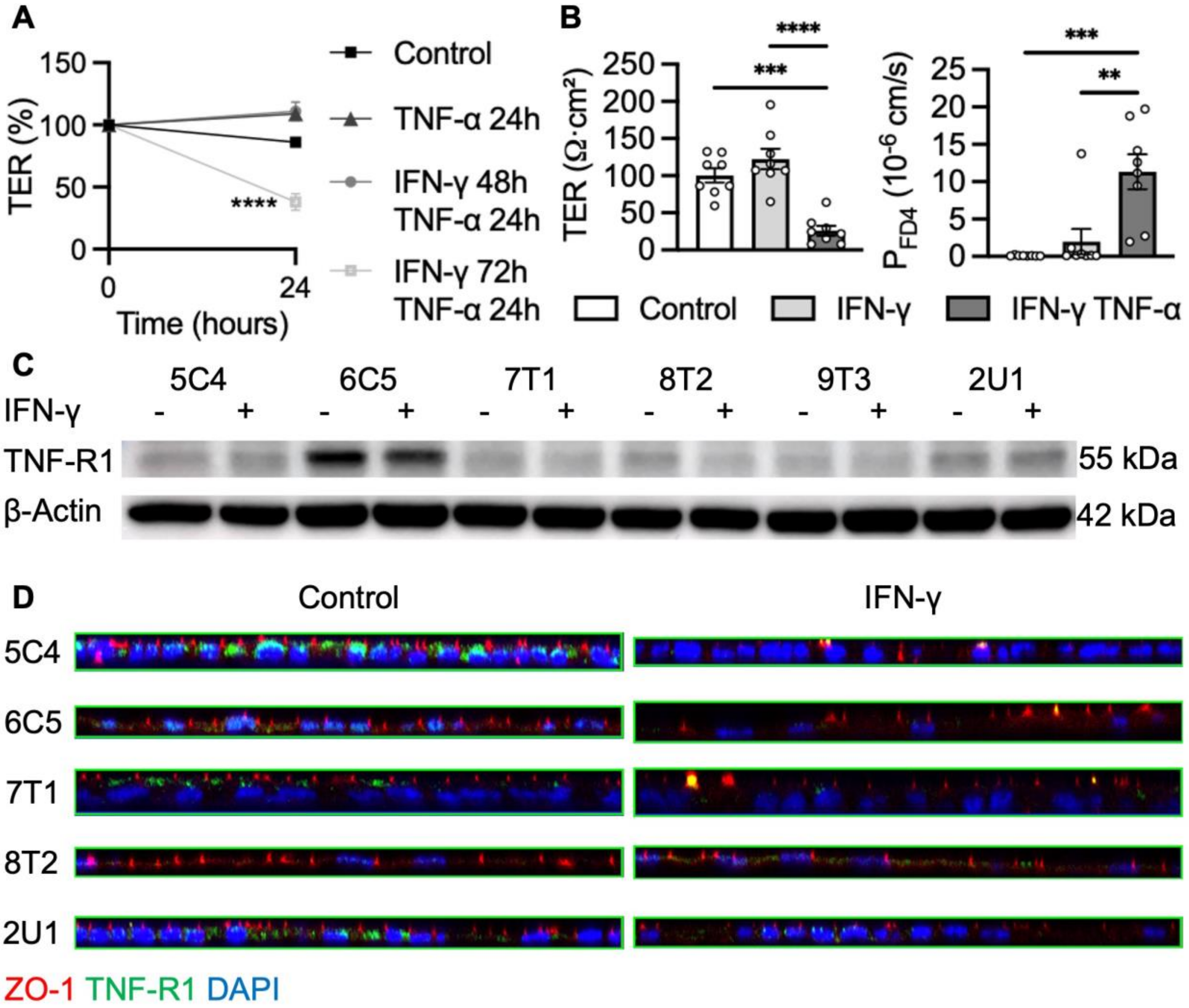

Figure S2. IFN-γ pre-stimulation. **(A)** Effects of 24h 100 ng/mL TNF-α stimulation following 1 ng/mL IFN-γ pre-stimulation on TER, n = 4. **(B)** TER and FD4 permeability following 72h 1 ng/mL IFN-γ pre-stimulation and 24h 100 ng/mL TNF-α stimulation, n = 8. Detection of TNF-R1 using **(C)** Western blots and **(D)** immunofluorescent staining (z-axes) of unstimulated control and 72h 1 ng/mL IFN-γ stimulated organoid monolayers. The merge of ZO-1 and TNF-R1 is shown in yellow.

References

1. VanDussen KL, Sonnek NM, Stappenbeck TS. L-WRN conditioned medium for gastrointestinal epithelial stem cell culture shows replicable batch-to-batch activity levels across multiple research teams. Stem Cell Research. 2019;37:101430.

2. Kim K-A, Kakitani M, Zhao J, Oshima T, Tang T, Binnerts M, et al. Mitogenic influence of human R-spondin1 on the intestinal epithelium. Science. 2005;309(5738):1256-9.

3. Heijmans J, de Jeude JFvL, Koo B-K, Rosekrans SL, Wielenga MC, van de Wetering M, et al. ER stress causes rapid loss of intestinal epithelial stemness through activation of the unfolded protein response. Cell reports. 2013;3(4):1128-39.
